# Generating patient‐matched 3D‐printed pedicle screw and laminectomy drill guides from Cone Beam CT images: Studies in ovine and porcine cadavers

**DOI:** 10.1002/mp.15681

**Published:** 2022-05-06

**Authors:** Andrew Kanawati, Alex Constantinidis, Zoe Williams, Ricky O'Brien, Tess Reynolds

**Affiliations:** ^1^ Westmead Hospital Sydney NSW Australia; ^2^ Faculty of Medicine and Health University of Sydney Sydney NSW Australia

**Keywords:** Cone Beam CT, 3D‐printing, spine

## Abstract

**Background:**

The emergence of robotic Cone Beam Computed Tomography (CBCT) imaging systems in trauma departments has enabled 3D anatomical assessment of musculoskeletal injuries, supplementing conventional 2D fluoroscopic imaging for examination, diagnosis, and treatment planning. To date, the primary focus has been on trauma sites in the extremities.

**Purpose:**

To determine if CBCT images can be used during the treatment planning process in spinal instrumentation and laminectomy procedures, allowing accurate 3D‐printed pedicle screw and laminectomy drill guides to be generated for the cervical and thoracic spine.

**Methods:**

The accuracy of drill guides generated from CBCT images was assessed using animal cadavers (ovine and porcine). Preoperative scans were acquired using a robotic CBCT C‐arm system, the Siemens ARTIS pheno (Siemens Healthcare, GmbH, Germany). The CBCT images were imported into 3D‐Slicer version 4.10.2 (www.slicer.org) where vertebral models and specific guides were developed and subsequently 3D‐printed. In the ovine cadaver, 11 pedicle screw guides from the T1–T5 and T7–T12 vertebra and six laminectomy guides from the C2–C7 vertebra were planned and printed. In the porcine cadaver, nine pedicle screw guides from the C3–T4 vertebra were planned and printed. For the pedicle screw guides, accuracy was assessed by three observers according to pedicle breach via the Gertzbein–Robbins grading system as well as measured mean axial and sagittal screw error via postoperative CBCT and CT scans. For the laminectomies, the guides were designed to leave 1 mm of lamina. The average thickness of the lamina at the mid‐point was used to assess the accuracy of the guides, measured via postoperative CBCT and CT scans from three observers. For all measurements, the intraclass correlation coefficient (ICC) was calculated to determine observer reliability.

**Results:**

Compared with the planned screw angles for both the ovine and porcine procedures (*n* = 32), the mean axial and sagittal screw error measured on the postoperative CBCT scans from three observers were 3.9 ± 1.9° and 1.8 ± 0.8°, respectively. The ICC among the observes was 0.855 and 0.849 for the axial and sagittal measurements, respectively, indicating good reliability. In the ovine cadaver, directly comparing the measured axial and sagittal screw angle of the visible screws (*n* = 14) in the postoperative CBCT and conventional CT scans from three observers revealed an average difference 1.9 ± 1.0° in axial angle and 1.8 ± 1.0° in the sagittal angle. The average thickness of the lamina at the middle of each vertebra, as measured on‐screen in the postoperative CBCT scans by three observes was 1.6 ± 0.2 mm. The ICC among observers was 0.693, indicating moderate reliability. No lamina breaches were observed in the postoperative images.

**Conclusion:**

Here, CBCT images have been used to generate accurate 3D‐printed pedicle screw and laminectomy drill guides for use in the cervical and thoracic spine. The results demonstrate sufficient precision compared with those previously reported, generated from standard preoperative CT and MRI scans, potentially expanding the treatment planning capabilities of robotic CBCT imaging systems in trauma departments and operating rooms.

## 1 INTRODUCTION

Pedicle screw posterior fixation is one of the most common spinal surgical procedures, treating conditions ranging from traumatic spine injury, infections, deformities (scoliosis and kyphosis), degeneration, and tumors (benign and malignant).^1^, ^2^, ^3^ Despite its prevalence, pedicle screw insertion is an inherently risky procedure. Accurate placement of each pedicle screw is paramount, with malposition potentially leading to damage of the spinal cord, nerve roots, or blood vessels, resulting in severe neural and vascular complications.^4^, ^5^ Perforation rates have been reported as high as 55% in the thoracic spine,^6^ with the majority of procedures performed using free‐hand techniques. Free‐hand techniques are heavily reliant on the experience of the surgeon, requiring extensive knowledge of anatomical landmarks and the orientation of the vertebrae.

Advanced medical imaging, both intraoperative and preoperative, provides a pathway for reducing the perforation rate. Intraoperatively, using 2D and 3D imaging (when available) has been shown to considerably lower perforation rates compared with using free‐hand techniques (5–13% for 2D imaging/fluoroscopy and 8–11% for 3D navigation^6^, ^7^, ^8^, ^9^, ^10^). However, the added radiation exposure to the patient and surgical personnel from these intraoperative image guided approaches must be seriously considered. Preoperatively, 3D images acquired using modalities such as computed tomography (CT) and magnetic resonance imaging (MRI) are frequently employed for preprocedural planning. Of growing interest in recent years is using preoperative 3D images to develop and 3D‐print patient specific pedicle screw drill guides (also referred to in the literature as templates and jigs), which will complement surgeon experience, rather than replace traditional techniques, and alleviate the reliance on intraoperative imaging.^11^, ^12^

3D‐printed patient‐specific pedicle screw drill guides allow individual anatomical characteristics to be taken into consideration, preplanning the screw trajectory and length for each vertebral level. To date, 3D‐printed pedicle screw drill guides from preoperative CT scans have been used clinically for the cervical and thoracic spine, with up to 98.5% accuracy (801 out of 813 pedicle screws without cortical violation) reported.^13^ Additionally, combined patient‐specific 3D‐printed pedicle screw and laminectomy guides have been investigated in human cadavers.^14^

CT is currently the modality of choice for developing 3D‐printed pedicle screw guides. Recently, there has been an emergence of robotic Cone Beam Computed Tomography (CBCT) imaging systems in trauma, providing additional 3D imaging capabilities to supplement conventional 2D fluoroscopic imaging for examination, diagnosis, and treatment planning of musculoskeletal injuries. To date, trauma in the extremities (i.e., wrist, ankle and knee) have been the primary focus of examination, diagnosis, and treatment planning from robotic CBCT imaging systems.^15^, ^16^, ^17^ However, spine injuries could also potentially be examined, diagnosed, and planned from CBCT images. These images could be acquired in trauma departments or even intraoperatively during initial surgery to stabilize the patient where musculoskeletal trauma will be treated in follow‐up surgeries. Here, whether CBCT images can be used during the treatment planning process in spinal instrumentation and laminectomy procedures, allowing accurate 3D‐printed drill guides to be generated for the cervical and thoracic spine is examined.

## 2 MATERIALS AND METHODS

**FIGURE 1:**
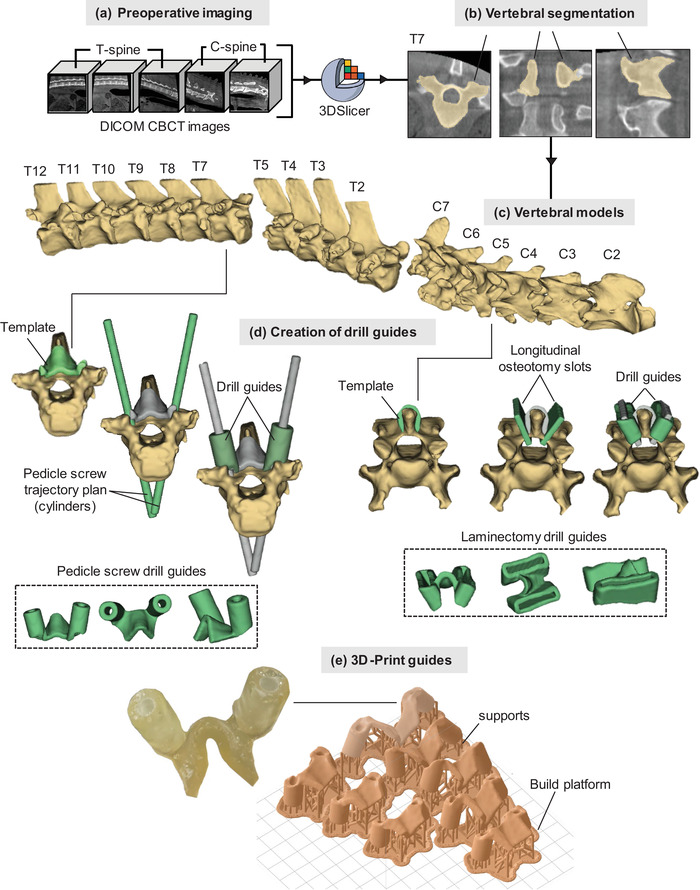
Development process for 3D‐printed drill guides from CBCT images. (a) Preoperative CBCT imaging. (b) Vertebral segmentation in 3D‐slicer. (c) Developing the vertebral models. (d) Creation of drill guides (pedicle and laminectomy). (e) 3D‐print guides

The accuracy of patient‐matched 3D‐printed pedicle screw and laminectomy guides generated from CBCT images was assessed using ovine and porcine cadavers, as depicted Figure 1. The cadavers used in this study were provided by the Laboratory Animal Services at the University of Sydney, in accordance with the University's Animal Ethics Procedures. Ethics to sacrifice the animals had been obtained through the University's Animal Ethics Committee for two previous, separate studies.

### 2.1 Preoperative imaging

The animal cadavers were imaged using the clinically available *syngo* DynaCT Body protocol on the Siemens ARTIS pheno (Siemens Healthcare, GmbH, Germany) robotic CBCT C‐arm system. The 3D field of view of the *syngo* DynaCT Body protocol is limited to 17 cm. Therefore, for the ovine cadaver, five planning scans were acquired to capture from C1 to T12. For the porcine cadaver, two planning scans were acquired to capture from C4 to T7. The *syngo* DynaCT Body protocol acquires 397 individual X‐ray projections evenly spaced over a 200° arc around the subject in 5 s. The 3D images were reconstructed using the in‐built software on the ARTIS pheno system, and exported to an external hard drive for processing, segmentation, and creation of the patient‐specific guides.

### 2.2 Image processing, segmentation, and creation of patient‐matched guides

Patient‐matched drill guides for each vertebra were constructed by a spine surgeon (A. K.), using 3D‐Slicer version 4.10.2 (www.slicer.org).^18^

Patient‐matched ovine pedicle screw drill guides were created for T1‐T5 and T7‐T12 and patient‐matched porcine pedicle screw drill guides were created for C4‐T7. A patient‐matched pedicle screw drill guide for the T6 vertebra of the ovine cadaver was not created as T6 was not visible in the planning images, falling in the overlap of two successive CBCT planning scans. To create the pedicle screw drill guides, first, a vertebral model was created using the semiautomated “grow from seeds” extension in the Segment Editor of 3D‐Slicer, Figure 1(b), as has been previously documented.^14^ The next step involved making a 4 mm hollow shell from the vertebral model. This shell was trimmed to create the template that rested on the spinous process and lamina (Figure 1(d)). The pedicle width was measured to plan the screw width. A 4.2 mm wide cylinder was created and placed in the ideal trajectory of the pedicle screws. The cylinder diameter was made approximately 80% of the narrowest cross‐sectional diameter of the pedicle.^19^ Cylinders, rather than screws with threads, were used to plan the screw trajectory to allow accurate and direct visualization of potential breaches, in different planes, on the 2D and 3D reconstructions. The ideal trajectory was judged by the cylinder being in the center of the pedicle in all three planes, completely inside the pedicle if possible. If the pedicle diameter was smaller than the smallest available screw (4.5 mm), planned in‐out‐in approaches were created, with the cylinder placed adjacent to the medial pedicle wall. Safe screw length was measured by using the distance between the entry point and anterior vertebral body surface, in line with the cylinder. Around the planned trajectory of the pedicle screws, drill guides with thickness of 4 mm and length of 10 mm were created, allowing the burr, set to a 14 mm depth for the laminectomy, to decorticate the entry point and to perform the laminectomy without changing burr handle settings (Figure 1(d)). The drill guide was then joined to the template that rested on the lamina and spinous process to create the final pedicle screw drill guide. The large amount of surface contact of the final pedicle screw drill guide with the bone ensured guide stability with gentle downward finger pressure during surgical steps.

Patient‐matched laminectomy drill guides for the ovine cadaver were created for C3–C7. As with the pedicle screw guides, the first steps were to create vertebral models using the semiautomated “grow from seeds” extension in the Segment Editor of 3D‐slicer and making a 4 mm hollow shell trimmed to create the template that rested on the spinous process and lamina (Figure 1(d)). Longitudinal lamina osteotomy slots were placed in the appropriate position to simulate preservation of the fact joints and pars interarticularis; however, the position of these guides could easily be tailored to surgeon preference. The slots were 3.5 mm wide, to accommodate a 3.5 mm round burr, and spanned the length of the lamina. The dorsal contour of the laminar osteotomy slot was created to match the deep surface of the lamina, set at a depth of 14 mm. This theoretically ensures that the burr tip will travel along the dorsal aspect of the ligamentum flavum and dura, if the burr tip is offset 14 mm from the handle piece. The laminectomy slots were then joined to the template that rested on the lamina and spinous process to create the final laminectomy guide.

### 2.3 3D‐printing of patient‐matched guides

The patient‐matched pedicle screw guides and laminectomy guides, as well as the vertebral biomodel files, were imported into Formlabs Preform software version 3.0.2 (Formlabs Inc., Sommerville, MA, USA) to be printed using Formlabs Form 2 (Formlabs Inc.) desktop 3D printer using Surgical Guide resin (Formlabs Inc.). The patient‐matched guides were oriented on the build platform to avoid placing supports on parts of the templates that would be in contact with the bone. The *z*‐axis layer thickness was set to 0.1 mm. The Surgical Guide resin is fully biocompatible and can be steam sterilized using an autoclave, allowing it to be used safely in the clinical setting. After printing, the models were soaked in 99% isopropyl alcohol for 20 min, and then UV cured for 30 min at 60°C in the Form Cure machine (Formlabs Inc.). After removal of supports, the models were inspected to exclude printing failures or errors. No additional postprocessing was required. An example of the 3D‐printed patient‐matched guide for the T7 (pedicle screw) vertebra for the ovine cadaver is provided in Figure 1(e).

### 2.4 Testing of patient‐matched guides: Pedicle screw

The patient‐matched 3D‐printed screw guides were used to place a total of 36 screws in the cervical and thoracic spine of an ovine (T1–T12) and a porcine (C4–T7) cadaver.

For both procedures, the cadavers were placed in a prone position. A midline posterior incision and approach was utilized. A subperiosteal dissection was performed for all the involved vertebrae. The areas where the planned guides were to be positioned were cleaned of soft tissues, with care taken not to disturb facet joint capsule and bone. Stability of the drill guides was confirmed with gentle downward digital pressure. A 4.2 mm drill was used to create a pilot hole for the pedicle screws. A ball‐tipped pedicle probe was used to confirm no palpable breach. The templated Evolution Surgical (Sydney, Australia) pedicle screw was then inserted.

Following the procedure, the placement of the pedicle screws was examined by CBCT (ovine and porcine cadaver) and CT (ovine cadaver only). The accuracy of the screw insertion was evaluated using the Gertzbein‐Robbins grading system and via calculating the mean axial and sagittal screw error, measured using an onscreen protractor^20^ from three observers (two experts, one nonexpert). For all measurements, the intraclass correlation coefficient (ICC)^21^ was calculated to determine observer reliability.

### 2.5 Testing of patient‐matched guides: Laminectomy cuts

The patient‐matched 3D‐printed laminectomy guides were used to perform 10 laminectomy cuts in the cervical spine of an ovine cadaver.

The ovine cadaver was placed in the prone position. A midline posterior incision and approach was utilized. A subperiosteal dissection was performed of all the involved vertebrae. The areas where the planned guides were to be positioned were cleaned of soft tissues. Stability of the drill guides was confirmed with gentle downward digital pressure. A 3.5 mm round‐tipped burr was used to thin the bone through the laminectomy guide. The burr length was set to 14 mm; however, the template was designed to allow 1 mm of bone in the base of the laminectomy trough, to allow accurate postoperative imaging assessment of the depth‐stop design of these guides. The average thickness of the lamina at the mid‐point of the lamina, in the cranial‐caudal direction, was used to assess the accuracy of the guides, measured via postoperative CBCT and CT scans from three observers (two experts, one nonexpert). For all measurements, the ICC was calculated to determine observer reliability.^21^

## 3 RESULTS

### 3.1 Pedicle screw insertion

**FIGURE 2:**
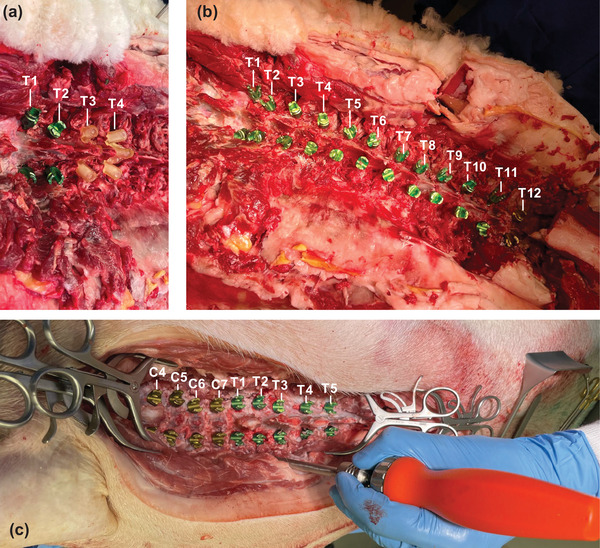
Images from the pedicle screw procedures in the ovine and porcine cadavers. (a) Ovine cadaver showing insertion of pedicle screws at T1 and T2 along with the patient‐matched 3D‐printed guides for T3 and T4 securely resting on the spinous process. (b) Ovine cadaver showing the insertion of all screws from T1 through to T12. (c) Porcine cadaver showing the insertion of all screws from C4 through to T5

In total, 42 screws were inserted with the aid of patient‐matched 3D‐printed screw guides in two procedures involving ovine and porcine cadavers. In the ovine cadaver, 24 pedicle screws were placed in 12 thoracic vertebrae (T1–T12). In the porcine cadaver, 18 pedicle screws were placed in nine vertebrae (C4–T5). Images of the pedicle screw placement during both procedures are provided in Figure 2.

Starting with the ovine cadaver, five vertebra levels (corresponding to 10 pedicle screws) were excluded from postoperative analysis due to either guide instability (T1) or misplaced guides (T6–T9 inclusive). The lamina footprint of the T1 guide was too small and was unstable when placed on the spinous process. Screw insertion for this level was abandoned during the procedure for this reason. A pedicle screw template for the T6 vertebrae was not created as the T6 was not visible in the planning images, falling in the overlap of two consecutive images. However, during the procedure, the guide for T7 was accidently placed on the T6 vertebra and pedicle screws inserted. This mistake was not corrected until T10, resulting in the wrong guides being used for the T6, T7, T8, and T9 levels.

**FIGURE 3:**
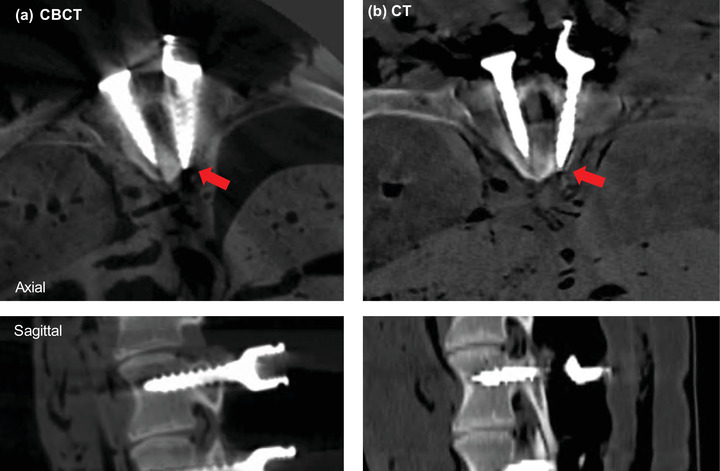
Images of the T9 vertebra from the ovine cadaver following the pedicle screw procedure using (a) CBCT and (b) CT imaging with metal artifact reduction reconstruction. Red arrows indicate location of cortical breach

For the remaining levels in the ovine cadaver, the accuracy of the guides was assessed according to pedicle breach as well as the mean axial and sagittal screw error via postoperative CBCT and CT scans. The postoperative CBCT images revealed four cortical breaches, which were evaluated using the Gertzbein–Robbins grading system. The breaches occurred at T1 Left (Grade E), due to guide instability, and T6 left (Grade C), T7 Left (Grade B), and T9 Right (Grade C), due to misplaced guides. An example of the 3D reconstructed images following the pedicle screw procedure in the ovine cadaver from in‐room CBCT, and conventional CT, displaying the breach at the T9 vertebra are provided in Figure 3. An additional cortical breach was identified at T12 in the postoperative CT. This breach was not observed on the postoperative CBCT images as it was outside the field of view.

The planned and measured (three observers) axial and sagittal angles from postoperative CBCT and CT images of the pedicle screws correctly placed and visible (T2–T5, T10–T11) in the ovine cadaver are provided in Tables 1 and 2, respectively. From the postoperative CBCT images, the average difference between the planned and measured screw angles from the three observers was 2.9 ± 1.6° in axial angle and 1.6 ± 0.8° in the sagittal angle. For the axial angle measurements, the ICC among all observers was 0.924, indicating excellent reliability.^21^ Similarly, for the sagittal angle measurements, the ICC among all observers was 0.822, indicating good reliability.^21^

**TABLE 1:** Planned and measured axial angles from postoperative CBCT and CT images of the pedicle screws placed in the ovine cadaver from three observers

	CBCT				CT		
Level	Planned	Observer 1	Observer 2	Observer 3	Observer 1	Observer 2	Observer 3
T2R	16.0°	12.9°	8.2°	11.3°	13.6°	11.4°	12.8°
T2L	34.6°	36.7°	31.8°	35.0°	38.7°	30.1°	37.5°
T3R	18.2°	14.8°	10.4°	14.0°	12.8°	9.6°	13.2°
T3L	24.8°	22.3°	24.0°	21.5°	25.1°	28.5°	24.3°
T4R	8.8°	12.4°	7.6°	11.8°	10.2°	11.5°	11.1°
T4L	25.2°	20.4°	23.6°	24.3°	20.6°	22.4°	19.7°
T5R	11.6°	11.4°	10.7°	10.8°	10.8°	11.5°	10.9°
T5L	15.4°	12.2°	15.0°	12.0°	14.0°	16.8°	14.8°
T9R	12.4°	8.8°	8.8°	10.2°	8.2°	12.3°	8.2°
T9L	16.6°	14.2°	16.0°	13.9°	15.0°	15.0°	15.0°
T10R	18.4°	15.8°	16.4°	13.8°	14.2°	19.1°	14.1°
T10L	15.7°	16.1°	18.9°	16.3°	14.7°	16.9°	13.7°
T11R	13.4°	19°	23.3°	17.8°	16.4°	23.8°	14.3°
T11L	23.0°	27°	21.3°	24.7°	24.0°	25.3°	23.2°

**TABLE 2:** Planned and measured sagittal angles from postoperative CBCT and CT images of the pedicle screws placed in the ovine cadaver from three observers

	CBCT				CT		
Level	Planned	Observer 1	Observer 2	Observer 3	Observer 1	Observer 2	Observer 3
T2R	2.0°	0.0°	0.0°	0.0°	0.0°	0.0°	1.2°
T2L	11.0°	11.6°	4.4°	12.0°	5.0°	8.0°	5.0°
T3R	0.0°	2.5°	1.5°	1.8°	0.0°	0.0°	0.0°
T3L	5.0°	5.8°	6.5°	5.8°	7.0°	5.8°	3.5°
T4R	9.0°	6.5°	8.2°	7.2°	8.0°	7.0°	7.6°
T4L	9.0°	9.4°	8.5°	9.7°	8.0°	8.7°	9.1°
T5R	5.4°	5.0°	7.5°	4.8°	6.9°	3.9°	6.2°
T5L	6.0°	5.7°	7.4°	5.3°	7.0°	6.4°	6.0°
T9R	5.0°	5.0°	7.5°	5.0°	4.0°	3.3°	3.8°
T9L	5.0°	5.0°	6.3°	5.0°	4.0°	6.1°	4.9°
T10R	8.0°	9.4°	9.7°	10.3°	5.0°	8.3°	3.5°
T10L	9.0°	9.0°	8.0°	11.2°	6.0°	9.9°	5.2°
T11R	5.3°	3.6°	5.7°	3.8°	4.0°	4.7°	5.0°
T11L	5.0°	0.0°	0.0°	0.0°	0.0°	0.0°	0.0°

From the postoperative CT images, the average difference between the planned and measured screw angles from the three observers was 2.7 ± 1.4° in axial angle and 1.7 ± 0.7° in the sagittal angle. For the axial angle measurements, the ICC among observers was 0.899, indicating good reliability.^21^ Similarly, for the sagittal angle measurements, the ICC among observers was 0.807, indicating good reliability.^21^

Directly comparing the measured axial and sagittal screw angles of the 14 pedicle screws from the postoperative CBCT and CT images revealed an average difference of 1.9 ± 1.0° in axial angle and 1.8 ± 1.0° in the sagittal angle respectively from the three observers.

In the porcine cadaver, all levels were included in the analysis with no guide instabilities or misplaced guides reported during the procedure. There were no cortical breaches observed for the procedure in the porcine cadaver. The planned and measured (three observers) axial and sagittal angles from postoperative CBCT images of the pedicle screws placed in the porcine cadaver are provided in Tables 3 and 4, respectively.

**TABLE 3:** Planned and measured axial angles from postoperative CBCT images of the pedicle screws placed in the porcine cadaver from three observers

Level	Planned (CBCT)	Observer 1	Observer 2	Observer 3
C3R	14.5°	14.2°	14.7°	12.8°
C3L	6.5°	5.2°	6.2°	6.9°
C4R	21.4°	0.0°	0.0°	0.0°
C4L	13.3°	11.0°	13.2°	8.5°
C5R	12.3°	9.0°	11.1°	10.9°
C5L	10.6°	4.0°	12.0°	0.0°
C6R	7.6°	7.0°	11.2°	4.8°
C6L	9.1°	4.0°	6.5°	0.0°
C7R	7.7°	7.0°	11.5°	4.8°
C7L	6.0°	5.0°	7.8°	9.4°
T1R	15.0°	6.0°	16.0°	4.2°
T1L	17.3°	6.0°	16.7°	9.6°
T2R	12.1°	4.0°	11.6°	0.0°
T2L	15.0°	12.0°	12.8°	9.9°
T3R	9.3°	4.0°	5.5°	0.0°
T3L	7.7°	8.0°	11.2°	5.0°
T4R	9.0°	0.0°	4.5°	0.0°
T4L	5.3°	2.0°	7.8°	2.0°

**TABLE 4:** Planned and measured sagittal angles from postoperative CBCT images of the pedicle screws placed in the porcine cadaver from three observers

Level	Planned (CBCT)	Observer 1	Observer 2	Observer 3
C3R	0.0°	0.0°	0.0°	0.0°
C3L	0.0°	0.0°	0.0°	0.0°
C4R	4.0°	0.0°	0.0°	0.0°
C4L	0.0°	0.0°	0.0°	0.0°
C5R	0.0°	0.0°	0.0°	0.0°
C5L	0.0°	3.0°	3.0°	3.5°
C6R	5.0°	0.0°	3.0°	0.0°
C6L	6.0°	6.0°	3.3°	3.5°
C7R	0.0°	2.0°	0.0°	4.0°
C7L	0.0°	0.0°	0.0°	0.0°
T1R	4.0°	0.0°	3.7°	4.0°
T1L	0.0°	0.0°	3.6°	4.0°
T2R	0.0°	2.0°	3.8°	4.0°
T2L	0.0°	6.0°	6.4°	4.0°
T3R	4.0°	7.0°	5.1°	4.0°
T3L	0.0°	6.0°	5.9°	8.0°
T4R	0.0°	0.0°	0.0°	0.0°
T4L	0.0°	4.0°	3.8°	4.0°

Comparing the planned axial and sagittal screw angles from the preoperative CBCT with the measured axial and sagittal screw angles from the postoperative CBCT images for the 18 pedicle screws from the C4 to T5 revealed an average difference of 4.9 ± 2.4° in axial angle and 2.2 ± 0.8° in the sagittal angle. For the axial angle measurements, the ICC among all observers was 0.488, indicating poor reliability,^21^ whereas for the sagittal angles, the ICC among all observers was 0.760, indicating good reliability.^21^

Across all pedicle screw insertions (ovine and porcine, totaling 32 pedicle screws), the average difference between the planned and measured angles from all observers was 3.9 ± 1.9° in axial angle and 1.8 ± 0.8° in the sagittal angle respectively. Across all axial angle measurements, the ICC among all observers was 0.855, and across all sagittal angle measurements, the ICC among all observers was 0.849, indicating good reliability in both cases.^21^

### 3.2 Laminectomy cuts

**FIGURE 4:**
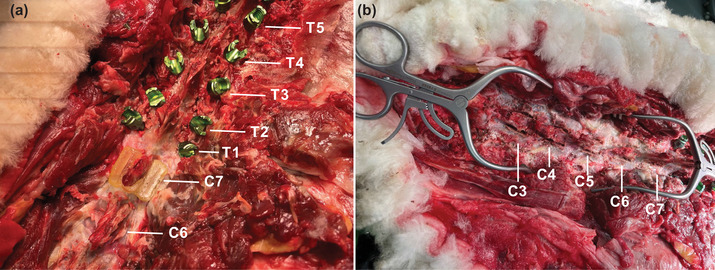
Images of (a) the cervical spine prior to laminectomy cuts with the 3D‐printed guide for the C7 vertebra resting on the spinous process and (b) following the laminectomy on the C3–C7 cuts in the ovine cadaver

In total, 10 laminectomy cuts with the aid of patient‐matched 3D‐printed laminectomy guides in an ovine cadaver from C3 to C7 were made. Images of one of the 3D‐printed laminectomy guides during the procedure and cuts post procedure are provided in Figure 4.

**FIGURE 5:**
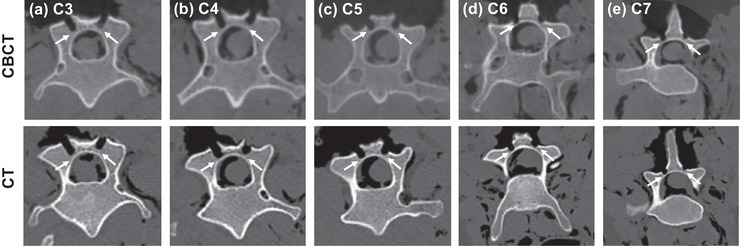
Postoperative CBCT (top) and CT (bottom) images of the cervical vertebra (C3–C7 (a)–(e), axial view) following the procedure. White arrows indicate the remaining lamina

Example of the postoperative CBCT images with on‐screen measurements of the remaining lamina thickness at the middle of each of the five vertebrae from Observer 1 are provided in Figure 5. The measurements of the remaining lamina thickness at the middle of each vertebra following laminectomy cuts on the ovine cadaver from both postoperative CBCT and CT scans from three observers are provided in Table 5. The average thickness of the mid‐point of the lamina, in the cranial‐caudal direction, as measured by three observers, on the postoperative CBCT scans over the five vertebrae was 1.6 ± 0.2 and 1.5 ± 0.2 mm with the postoperative CT. The ICC among observers was 0.693 for the postoperative CBCT scans, indicating moderate reliability and 0.866 for the postoperative CT scans, indicating good reliability.^21^ No lamina breaches were observed in either the CBCT or CT postoperative images.

**TABLE 5:** Measurements of the remaining lamina thickness at the middle of each vertebra following laminectomy cuts on the ovine cadaver from both postoperative CBCT and CT scans from three observers

	CBCT			CT		
Level	Observer 1	Observer 2	Observer 3	Observer 1	Observer 2	Observer 3
C3R	2.4 mm	1.9 mm	2.3 mm	2.2 mm	2.3 mm	2.5 mm
C3L	2.1 mm	1.7 mm	1.8 mm	2.0 mm	1.7 mm	1.9 mm
C4R	2.1 mm	2.0 mm	1.9 mm	1.9 mm	1.4 mm	1.7 mm
C4L	1.4 mm	1.2 mm	1.7 mm	1.4 mm	1.1 mm	1.5 mm
C5R	1.7 mm	1.3 mm	2.1 mm	1.5 mm	1.4 mm	1.1 mm
C5L	0.9 mm	0.9 mm	1.2 mm	0.9 mm	0.9 mm	1.1 mm
C6R	1.8 mm	1.4 mm	1.7 mm	1.4 mm	1.6 mm	1.6 mm
C6L	1.3 mm	0.7 mm	1.4 mm	0.9 mm	0.8 mm	1.1 mm
C7R	1.6 mm	2.2 mm	2.1 mm	2.0 mm	2.0 mm	2.4 mm
C7L	1.1 mm	1.1 mm	1.3 mm	1.1 mm	1.1 mm	1.4 mm

## 4 DISCUSSION

In this study, the effectiveness of using conventional CBCT scans to develop patient‐matched 3D‐printed pedicle screw drill and laminectomy guides was examined in ovine and porcine cadavers. The 3D‐printed guides enabled the procedures to be performed accurately, with cortical breaches only occurring during the pedicle screw procedure in the ovine cadaver due to unstable or misplaced guides. The results (measured mean axial and sagittal screw error for the pedicle screw placement and remaining lamina thickness for the laminectomies) demonstrated sufficient precision compared with those previously reported generated from standard preoperative CT and MRI scans,^14^, ^22^, ^23^, ^24^ potentially expanding the treatment planning capabilities of robotic CBCT imaging systems in trauma departments and operating rooms. Additionally, in the ovine cadaver, it was also shown that the in‐room postoperative CBCT provided acceptable assessment accuracy of the pedicel screw fixation and laminectomy procedures performed, matching that of standard postoperative CT.

Three observers (two experts and one nonexpert) completed the measurements (mean axial and sagittal screw angle for the pedicle screw placement and remaining lamina thickness for the laminectomies). For all the screw angle measurements, the three observers demonstrated good to excellent reliability as defined from the ICC. When considering only the two expert observers (observers 1 and 3 in Tables 1, 2, 3, 4), the reliability improved to excellent for all measurements, as defined from the ICC. Comparatively, for the remaining lamina thickness measurements, the reliability of all three observes was reduced to moderate to good, as defined from the ICC. The reduction is most likely due to the additional requirements to complete the measurements (i.e., identifying the middle of the vertebra and middle of the cut), potentially adding variability into each observers’ measurements.

Here, a fixed‐room robotic imaging system within the operating room for both the preoperative and postoperative planning and assessment was utilized. This represents the scenario where intraoperative imaging is available, and therefore images collected during the first procedure to stabilize the patient (e.g., controlling a intrathoracic hemorrhage) could be utilized to plan subsequent procedures to address the musculoskeletal trauma. This has the potential to eliminate the need for additional CT imaging (reducing overall dose received by the patient) and limit unnecessary transfer/handling of the patient for additional scans. However, it should be noted that intraoperative imaging is not universally available and therefore such a workflow can currently only be considered for facilities with the required clinical infrastructure. Alternatively, emerging systems such as the Siemens Multitom RAX robotic imager^17^, ^25^ or mobile CBCT systems, widely found outside of the operating theater in trauma departments, are more common and would be highly suited to generating the preoperative planning images for a broader group of patients.

Despite the success of the procedures performed, there were a number of limitations associated with the studies reported here. First, these studies used animal cadavers, meaning that there was no motion (i.e., breathing, beating heart) to contend with during the preoperative imaging. Motion artifacts in the planning scans can propagate errors throughout the planning process from segmentation, guide development to 3D‐printing. Future studies involving live animals will be used to quantify the extent of these errors and determine whether adaptive CBCT imaging approaches^26^, ^27^, ^28^ are required to reduce the size and number of motion artifacts. Another limitation in the planning process of these studies was that the segmentation software (3D Slicer) used is not United States Food and Drug Administration (US FDA) approved. 3D Slicer was selected for these studies due to it being a free, open‐source software. There are a number of US FDA approved software packages for segmentation; however, their high cost can be prohibitive of widespread use.

The third limitation was the small numbers of vertebrae examined. The number of vertebrae examined in the studies was limited by the combination of how long the cadavers could be stored between acquiring the planning scans and performing the procedure, availability of surgical personnel and the time required to design and print the guides. Future studies will look to scale‐up the 3D‐printing capabilities, allowing a larger number of guides to be printed simultaneously, reducing the time required between planning and procedure. An additional limitation was not having access to a surgical grade burr for the laminectomy procedure, potentially reducing the accuracy of the cuts. Future studies will look to use a surgical grade burr to ensure maximal precision in the depth of the laminectomy cuts can be achieved.

More generally, another potential drawback of using CBCT scans to develop 3D printed surgical guides is the limited field of view. In the ovine cadaver, for example, the planned procedures spanned 62.5 cm and required five conventional CBCT scans to capture the C1 through to T12 vertebrae. Unfortunately, the T6 vertebrae fell in the overlap between scan number 3 and 4, preventing clear visualization of the vertebrae. Therefore, it was not possible to create a pedicle screw guide for T6. In practice, this could have been rectified by acquiring an additional scan. However, due to the timing of the study and limited access to the cadaver, it was not possible to rescan the cadaver and print a new guide prior to the procedure taking place. If conventional CBCT scans are going to be used to develop 3D printed surgical guides in the future, care must be taken to ensure every vertebra within the required procedural field of view can be visualized. Alternative (i.e., noncircular) imaging trajectories^29^, ^30^ are also currently being investigated to help extend the CBCT imaging field of view. No approach with a fixed‐room robotic CBCT system has been implemented clinically; however, a real‐time implementation of multiturn reverse helical trajectory on a clinical fixed‐room robotic CBCT system has recently been investigated.^31^

## 5 CONCLUSION

Here, CBCT images have been used to generate accurate 3D‐printed pedicle screw and laminectomy drill guides for use in the cervical and thoracic spine. Further research, including studies in live animals, will be required to determine if generating 3D‐printed pedicle screw drill guides from CBCT images can be clinically viable.

## CONFLICT OF INTEREST

The authors have no conflicts to disclose.
